# Hyperbaric Oxygen Therapy as a Complementary Treatment in Glioblastoma—A Scoping Review

**DOI:** 10.3389/fneur.2022.886603

**Published:** 2022-07-01

**Authors:** Diogo Alpuim Costa, Mafalda Sampaio-Alves, Eduardo Netto, Gonçalo Fernandez, Edson Oliveira, Andreia Teixeira, Pedro Modas Daniel, Guilherme Silva Bernardo, Carla Amaro

**Affiliations:** ^1^Haematology and Oncology Department, CUF Oncologia, Lisbon, Portugal; ^2^NOVA Medical School (NMS), Faculdade de Ciências Médicas (FCM), Lisbon, Portugal; ^3^Faculty of Medicine, University of Lisbon, Lisbon, Portugal; ^4^Centro de Medicina Subaquática e Hiperbárica, Azinhaga dos Ulmeiros, Lisbon, Portugal; ^5^Centro Hiperbárico de Cascais, Cascais, Portugal; ^6^Faculty of Medicine, University of Porto, Oporto, Portugal; ^7^PTSurg – Portuguese Surgical Research Collaborative, Lisbon, Portugal; ^8^Radioncology Department, Instituto Português de Oncologia de Lisboa Francisco Gentil (IPOLFG), E.P.E., Lisbon, Portugal; ^9^Radioncology Department, CUF Oncologia, Lisbon, Portugal; ^10^Neurosurgery Department, Cluster CUF Descobertas, Lisbon, Portugal; ^11^Urology Department, Hospital Professor Doutor Fernando Fonseca, Amadora, Portugal; ^12^Otorhinolaryngology Department, CUF Descobertas, Lisbon, Portugal

**Keywords:** glioblastoma, glioma, hypoxia, cancer, radiation, chemotherapy, hyperbaric oxygenation, hyperbaric oxygen

## Abstract

Glioblastoma (GBM) is the most common and aggressive malignant brain tumor in adults. The mainstay of management for GBM is surgical resection, radiation (RT), and chemotherapy (CT). Even with optimized multimodal treatment, GBM has a high recurrence and poor survival rates ranging from 12 to 24 months in most patients. Recently, relevant advances in understanding GBM pathophysiology have opened new avenues for therapies for recurrent and newly diagnosed diseases. GBM's hypoxic microenvironment has been shown to be highly associated with aggressive biology and resistance to RT and CT. Hyperbaric oxygen therapy (HBOT) may increase anticancer therapy sensitivity by increasing oxygen tension within the hypoxic regions of the neoplastic tissue. Previous data have investigated HBOT in combination with cytostatic compounds, with an improvement of neoplastic tissue oxygenation, inhibition of HIF-1α activity, and a significant reduction in the proliferation of GBM cells. The biological effect of ionizing radiation has been reported to be higher when it is delivered under well-oxygenated rather than anoxic conditions. Several hypoxia-targeting strategies reported that HBOT showed the most significant effect that could potentially improve RT outcomes, with higher response rates and survival and no serious adverse events. However, further prospective and randomized studies are necessary to validate HBOT's effectiveness in the ‘real world' GBM clinical practice.

## Introduction

Glioblastoma (GBM) is the most common malignant primary brain tumor, representing more than 50% of all gliomas and ~20% of all primary malignant central nervous system (CNS) tumors (1–3). With an incidence rate of 3.19 cases per 100,000 individuals and a median age of 64 years, it is uncommon in children. The incidence is higher in men and Caucasians compared to Africans and Afro-Americans (4).

Currently, the standard of care consists of a multimodality treatment approach, including surgical resection, radiotherapy (RT), systemic therapy (chemotherapy—CT, targeted therapy), and supportive care. However, the overall prognosis remains unsatisfactory, long-term survival is rare, and the associated morbidity related to declining neurologic function and quality of life has a devastating impact on patients and their supportive social context (5, 6).

Blood vessels in GBM are dilated, tortuous, and excessively thin basement membrane. Subsequently, tumor vasculature is functionally abnormal with markedly increased interstitial fluid pressure, aggravating hypoxia and acidosis (7). Hence, under such conditions, conventional treatment is less effective because of the hypoxic microenvironment, contributing to a challenging scenario (8). Hypoxia emerges as an important factor involved in the tumor growth and aggressiveness, metabolic reprogramming, angiogenesis, resistance to cell death, immunosuppression, inflammation, and glial-to-mesenchymal transition of cancer cells. Thus, therapeutic approaches that target hypoxia-induced factors, such as a monoclonal antibody against VEGF (e.g., bevacizumab), have been used, but failed to increase survival (7, 9, 10).

A better understanding of hypoxia-mediated protection of GBM cells has led to other experimental types of treatment being tested in this setting, including hyperbaric oxygen therapy (HBOT). HBOT consists of breathing pure medical oxygen intermittently while inside a hyperbaric chamber pressurized to greater than sea level pressure (1 atmosphere absolute [ATA]). It is applied in several clinical conditions being also used for professional and military diving training. HBOT is considered therapeutic if the pressure used is 1.4 ATA or higher. Most series apply 2.0–2.5 ATA for 70 to 120 min (11, 12).

In 1996, Kohshi et al. published a pioneering report on the combination of HBOT with RT as a possible complementary treatment for patients with brain tumors (13). Several studies have substantiated the adjuvant use of HBOT with RT and CT from that moment on. In addition, HBOT has also proved to be useful in treating some cases of radiation-induced brain injury (14). Notwithstanding, scientific evidence is still needed to support these indications.

Given the lack of substantial data to incorporate HBOT as a complementary treatment in GBM, this review aims to comprise the existing literature and scientific evidence, analyze results from previous studies, and identify potential horizons that may merit further exploitation.

## Methodology

In order to include all available data on GBM and HBOT, we chose the scoping review as a methodology, as it could better map the existing literature and help to elucidate concepts.

Initially, our protocol did not contain any language or data limits. Our search strategies included case series, case reports, clinical practice recommendations, review articles, and supplemental files.

Queries were run on PUBMED, Google Scholar, and Cochrane. The first query employed “hyperbaric oxygenation” [MeSH] AND “brain” [MeSH] AND “neoplasms” [MeSH]. To ensure maximum inclusion, a second query was run on PUBMED with “glioma” [MeSH] AND “hyperbaric oxygenation” [MeSH].

This review followed the PRISMA criteria (15). Inclusion criteria consisted of (1) review articles of HBOT; (2) articles and expert meetings of clinical practice recommendations for GBM management; (3) articles regarding the physiological effects of oxygen on GBM; (4) case series of patients with GBM treated with HBOT; (5) case reports of patients with GBM treated with HBOT; (6) articles detailing preclinical data on the effects of oxygen on tumor cells; (7) articles/abstracts with available translations in Portuguese, English, Spanish, or French. The exclusion criteria comprised the following: (1) articles not mentioning GBM and/or HBOT at any point; (2) articles that only comprised normobaric oxygen use.

By the 27th of November 2021, our database search was completed.

## Results

**Figure 1:**
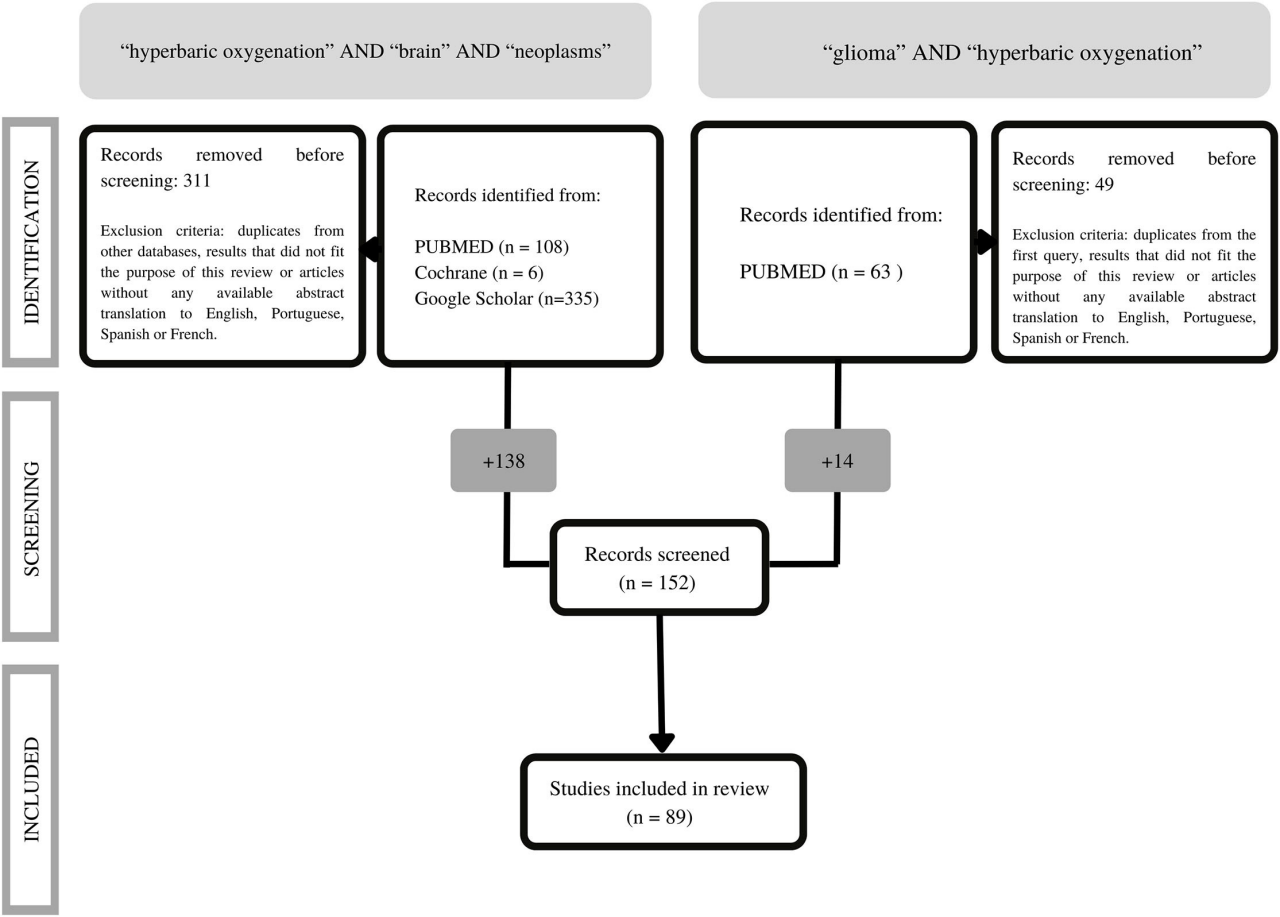
Adaptation of the PRISMA 2020 flow diagram for new systematic reviews [from (16)].

In the previously mentioned identification phase (e.g., article searching), we obtained 512 results, which are detailed in Figure 1.

After excluding findings with no available abstract in English, duplicates and records that did not suit the purpose of this review, a total of 152 articles entered the subsequent stage. Articles not written or without available translation into Portuguese, English, Spanish, or French were excluded in the screening phase.

Two separate investigators (DAC and MS-A) screened all articles independently throughout this stage, ultimately including 89 articles. Therefore, we considered all research that pertained to GBM and HBOT eligible.

## Discussion

### Glioblastoma

We have witnessed a gradual improvement in GBM's outcomes, presumably due to recent advances in multimodality therapy with surgery, new RT modalities, and anticancer agents (17). Although neuronavigation and intraoperative magnetic resonance (MRI) imaging are related to higher resection rates, they are not considered crucial prognostic factors in Neuro-Oncology (17).

Currently, the standard of care for these patients is maximal safe resection followed by postoperative RT with concomitant and adjuvant temozolomide (TMZ), as established by the phase III trial 22981/26981–EORTC/NCIC (1, 2). The median overall survival (OS) with this multimodal treatment is approximately 14 months (1, 2).

TMZ, an oral imidazotetrazinone methylation agent that inactivates the DNA repair enzyme O^6^-alkylguanine-DNA alkyltransferase, is the primary CT choice for glioma in clinical practice (9, 18). TMZ improves the patient's OS rate and extends progression-free survival (PFS) after surgery (1). However, the use of TMZ is limited due to its short plasma half-life, systemic toxicity, and limited access through the blood–brain barrier (9, 19). Moreover, the 5-year OS is still <8% under this treatment protocol (9).

The standard radiation dose is 60 Gy in 1.8–2.0 Gy per fraction administered over 6 weeks. Most dose-escalation studies were performed before the era of intensity-modulated RT (IMRT) (1, 2). IMRT allows for the selective delivery of high-dose per fraction radiation to the target volume while minimizing the dose to surrounding normal tissues (20). In addition, hypofractionation confers the benefits of shortening the RT course in patients with limited life expectancy, reducing costs and possibly increasing malignant cell death and decreasing accelerated repopulation (1, 20).

Panet-Raymond et al. (21) retrospectively studied the feasibility of delivering hypo-IMRT with concurrent and adjuvant TMZ in a cohort of 35 patients. The median OS was comparable to that after conventional fractionation, and the regimen was tolerable with no undue toxicity.

Helical tomotherapy (HT) can provide different modulation techniques and geometric flexibility compared to standard IMRT. In addition, superior control of dose distribution may allow for better dose uniformity within target and/or spare organs at risk (2).

Even though the optimal hypofractionation regimen using IMRT remains to be determined, the administration of hypo-IMRT with concomitant and adjuvant TMZ was found to be safe and feasible. The OS and PFS were comparable to those reported with conventional fractionation regimens (2).

Hypoxia has been suggested to contribute to glioma resistance to TMZ (9). Therefore, increasing the oxygen concentration in the tumor tissue may enhance CT effects (9). Likewise, the efficacy of ionizing radiation can be increased through the use of HBOT (22, 23) since the oxygen effect is caused by the reinforced formation of reactive oxygen species (ROS) and free radicals in the cell (23).

### Hypoxia

#### Hypoxia and Radioresistance

Reoxygenation during cancer treatment is one of the pinnacles of the four “Rs” of radiobiology, along with repair, repopulation, and reassortment (or redistribution) (24).

The presence or absence of molecular oxygen dramatically influences the biological effect of radiation in biological tissues. To generate an effect, molecular oxygen must be present during radiation exposure, or at least for the lifetime of the radiation-generated free radicals (24–26).

The primary mechanism of RT stems from the creation of ROS, consequently inducing cell death by apoptosis, necrosis, autophagy, and senescence. As oxygen is required for ROS generation, hypoxic tumors are resistant to the cytotoxic effects of RT. Hence, oxygen is responsible for the permanent damage induced by free radicals. Consequently, in its absence, these RT-induced lesions can be repaired.

For radiosensitization, only a small amount of oxygen is required. It is estimated that 0.5% oxygen (pO_2_ of about 3 mmHg) results in an intermediate radiosensitivity between hypoxia and total oxygenation. Several tumors implanted in animals have been shown to contain hypoxic cells that limit curability by single doses of X-rays. Hypoxic fractions range from 0 to 50%, with an average of about 15%.

There is strong evidence that human tumors contain hypoxic cells, including histological appearance, oxygen probe measurements, nitroimidazole binding, positron emission tomography (PET)/SPECT studies, and pretreatment hemoglobin levels. Oxygen probes with fast response times, implanted in a tumor and under computer control, may be used to obtain an oxygen profile. In addition, hypoxia in tumors can also be visualized with hypoxia markers such as pimonidazole or hypoxia-inducible factors (HIF—which play a role in radioresistance by upregulating downstream genes involved in apoptosis, metabolism, proliferation, and neovascularization) (27–32).

As a dynamic environment, an irradiated tissue can reoxygenate itself when hypoxic cells become, once more, oxygenated. Though the extent of reoxygenation and its rate varies widely for different experimental animal tumors, if it is rapid and complete, hypoxic cells will have little influence on the outcome of a fractionated radiation schedule.

While acutely hypoxic cells rapidly reoxygenate as the tumor's blood vessels open and close, chronically hypoxic cells respond slowly as the tumor shrinks. It has also been described that HBOT has a positive effect on radiosensitization in low-oxygenated tumor regions, as it helps to surpass the constraints of oxygen deficiency. Albeit reoxygenation cannot yet be measured in *in vivo* human tumors, it is presumed to occur.

#### Hypoxia and Chemoresistance

Relying on anaerobic metabolism, gliomas thrive under hypoxic microenvironments, contributing to their poor prognosis. In fact, high lactic acid concentrations correlate with a worse prognosis (33).

Hypoxia plays a critical role in maintaining glioma malignancy and resistance, as it induces HIF pathways responsible for regulating important cell cycle genes (34). Consequently, hypoxia will also contribute to cell stemness maintenance by inhibiting cell cycle progression (blocking cells in the G1 phase).

**Figure 2:**
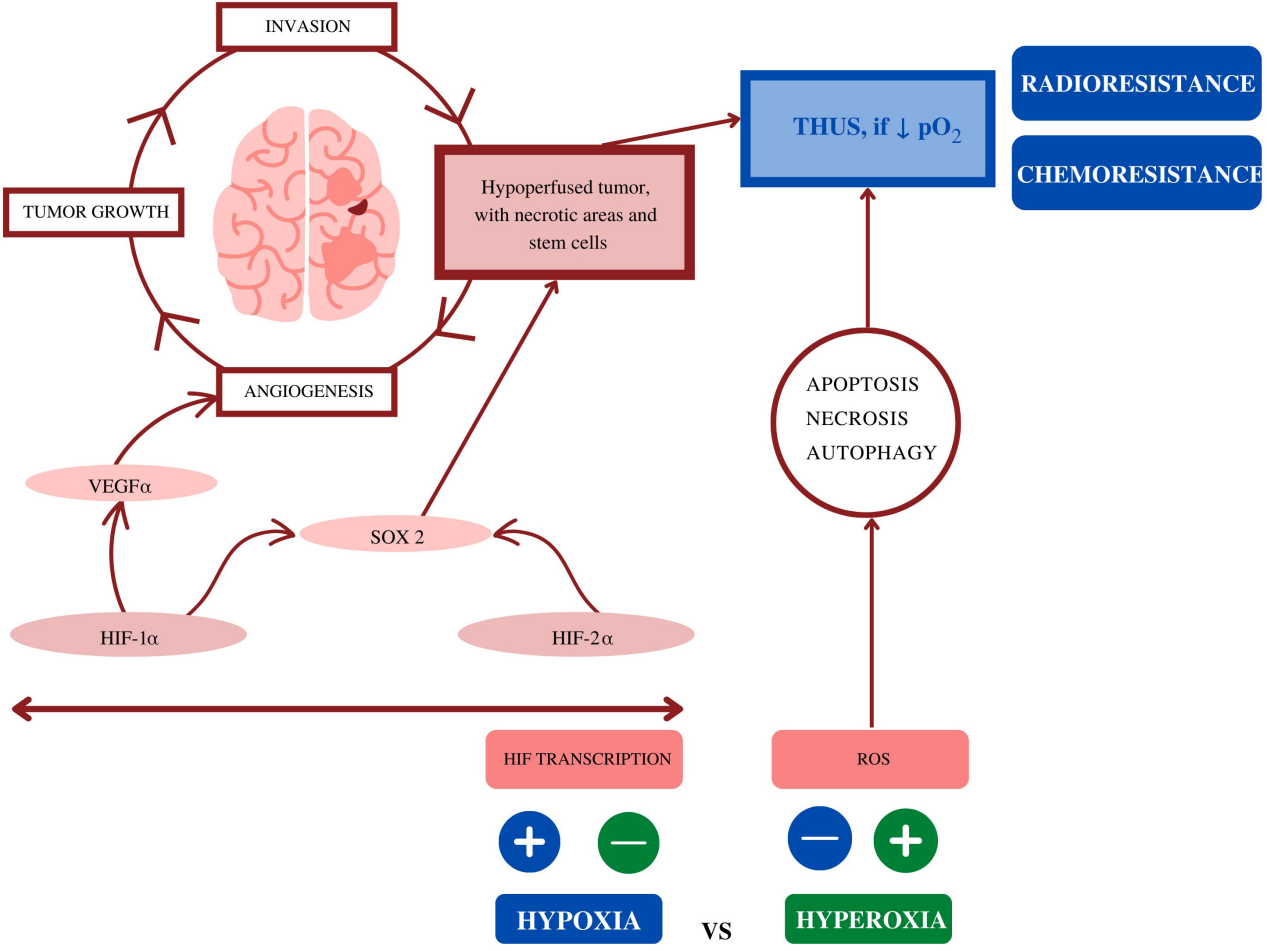
Overview of the influence of hypoxia on glioblastoma: Oxygen deficiency is the main contributor to both chemo- and radioresistance (made with Canva® 2022, under a *Pro* license). HIF, hypoxia-inducible factors; HIF-1α, hypoxia-inducible factor 1α; HIF-2α, hypoxia-inducible factor 2α; pO_2_, partial pressure of oxygen; ROS, reactive oxygen species; SOX2, sex-determining region Y-box 2.

Oxygen starvation mechanisms help explain GBM resistance to CT, promoting tumor growth, angiogenesis, and invasion. Angiogenesis is largely induced and driven by HIF-1α (35). However, the aberrant neovascularization that follows creates immature and flawed vessels that are unable to perfuse the rapidly growing tumor, leading to a hypoperfused neoplasm. The latter results in increasingly numerous necrotic areas (Figure 2).

After demonstrating that glioma cells submitted to hyperbaric oxygenation grow, Wang et al. (36) also studied the effects of HBOT on the expression of the two main molecules that contribute to tumor progression: HIF-1α and HIF-2α. Both are also responsible for regulating sex-determining region Y-box 2 (SOX2—a stem cell marker responsible for blocking the cell cycle, inducing and maintaining cell stemness). In the same way, HIF-1α promotes the multidrug resistance mutation 1 (MDR1) transcription, thus enhancing multidrug resistance to cytotoxic agents. The authors hypothesize that a more oxygen-rich microenvironment not only promotes cell cycle progression but also decreases cell stemness (correlated with chemoresistance) by inhibiting HIF-1α, HIF-2α, and SOX2. In their experiment, mice submitted to HBOT alone had increased tumor volumes than control mice (e.g., normoxia). Yet, when HBOT was used adjunctively with TMZ, the mice had smaller tumors and prolonged survival than those treated with TMZ alone or given only HBOT. Therefore, despite promoting cell cycle progression (and inevitably, tumor growth), HBOT as an adjuvant therapy contributes to chemosensitisation (36). Even with this seemingly contradictory result, it is relevant to emphasize that the available literature does not support the hypothesis of HBOT as a promoter of malignant cell proliferation. Malignancy is not a contraindication for HBOT (37).

In 2004, Evans et al. had already established an association between hypoxia and tumor aggressiveness in 18 patients with supratentorial glial neoplasms. By intravenous administration of the agent 2-nitroimidazole EF5 [2-(2-nitro-1-H-imidazole-1-yl)-N-(2,2,3,3,3-pen-tafluoropropyl) acetamide] in the 24 to 48 h before the tumor biopsy, they measured hypoxia levels *in vivo*. Their findings support that severe hypoxia correlates with more aggressive clinical behaviors, with early recurrence being one of the consequences (38).

Hadanny and Efrati (39) also proposed an interesting concept: the “hyperoxic–hypoxic paradox.” This is based on the notion that the metabolic changes experienced at the cellular level triggered by hypoxic environments can also be induced by intermittent exposure to hyperoxia. In these conditions, tissue regeneration is activated by stimulating sirtuin 1 (SIRT1) and mitochondrial transfer. This results in a neuroprotective effect and the induction of neuro-recovery mechanisms (39).

### Evidence Regarding Hyperbaric Oxygen Therapy

#### Principles and Fundamentals

The first documented use of hyperbaric medical therapy was in 1662 by Henshaw, a British physician who placed patients in a pressurized air container. Interestingly, it was carried out before the formulation of the Boyle–Mariotte Law, which described the relationship between pressure and volume of a gas, and before John Priestly discovered oxygen more than 100 years later.

Presently, the treatment of a patient resorting to HBOT occurs at elevated atmospheric pressures (higher than 1 ATA), with 100% oxygen. Thus, the tissue's oxygen partial pressure increases, resulting in a myriad of benefits, including improved oxygen supply, reduced inflammation and oedema, and even inhibition of infection.

HBOT employs several physiological principles of gas' diffusion and solubility, especially to predict the behavior of oxygen under pressure. Based on its solubility under pressure, when the oxygen concentration in a solution increases, the diffusion gradient for its delivery deeper into tissues also increases. Ultimately, the increase in dissolved oxygen generated by HBOT has several physiological effects capable of altering tissue responses to disease and injury. In a hyperbaric environment, it is possible to dissolve sufficient oxygen in the plasma to meet the body's usual needs. As physically dissolved oxygen will be more available than the one bound to hemoglobin, oxyhemoglobin will be able to pass unchanged from the arterial to the venous side (40).

The brain is the organ where HBOT has the most important metabolic effects. These are more pronounced in hypoxic/ischaemic states, where hyperbaric oxygenation can reduce cerebral oedema and improve the function of neurons rendered inactive by these lesion-inducing mechanisms. An enhanced electrical brain activity reflects this improvement in brain function. HBOT can also improve microcirculation, relieve cerebral hypoxia, partially preserve damaged tissue, and improve cerebral metabolism.

During treatment, the starting optimal pressure for patients with brain injury is 1.5 ATA, as the cerebral glucose metabolism is balanced at this pressure. Consequently, raising the pressure, even if only to 2 ATA, may have unfavorable effects. At high pressures, oxygen can have acute cytotoxic effects. These were first described in 1878 and usually manifest with seizures—the Paul-Bert effect—whose incidence ranges from 0.002 to 0.061% (41–43). Individual factors (e.g., previous seizures, carbon dioxide retention, alcohol, few drugs, fever, hypothermia, anxiety, cerebral trauma, metabolic acidosis, shock, increased pressure, and oxygen exposure) (44–46) can alter a person's sensibility and reduce their seizure threshold. The first-line approach for hyperoxic seizures is immediate treatment interruption.

Generally, HBOT therapy is safe and well-tolerated by humans at 1.5–2 ATA (11, 12).

#### Adverse Events

Although rare and usually non-severe, HBOT has known potential side effects. In a retrospective analysis of 1.5 million treatments, only 0.68% were associated with an adverse event, with barotrauma and confinement anxiety being the most reported events (42). When patients are previously studied and the correct protocol is applied, side effects are scarce.

HBOT acts by increasing pressure (via pure oxygen administration) on the patient. The most common adverse effects will occur by disturbing air cavities, including ear, paranasal sinus, pathologic dental spaces, and emphysematous bulla (47).

Middle ear barotrauma (MEB) is this therapy's most common side effect. Its incidence varies according to the series and ranges from 0.37 to 84% in non-ventilated patients vs. 94% in ventilated patients (42, 48). Clinical presentation of MEB usually includes ear pain, hearing loss, tinnitus, and, more rarely, vertigo. Diagnosis is performed by otoscopy, and treatment will depend on its severity, generally consisting of decongestants, nasal and systemic steroids, and antibiotics.

To correctly equalize the air in the middle ear, the eustachian tube (which connects the middle ear to the nasopharynx) needs to be opened by the patient using Valsalva maneuvers (e.g., swallowing or chewing). Nevertheless, in patients who have difficulties equalizing air pressure, even medicated and trained, in some cases, a myringotomy with ventilation tubes may be necessary.

Sinus barotrauma (SB) is the second most frequent adverse effect, usually associated with upper airway infection. SB is rare with controlled pressurization and depressurization (49). The manifestations will depend on the affected sinus. Clinical presentation may include sensations of pressure, epistaxis, and pain. Treatment includes nasal decongestants, antihistamines, and/or steroid nasal spray, which should be prescribed prior to HBOT for SB prevention (50).

#### Hyperbaric Oxygen Therapy as a Hypoxia Reverser Treatment

Despite being a highly vascularized tumor, GBM is characterized by extensive necrotic areas with surrounding hypoxia (6). Hypoxia has been associated with an increase in glioma stem-like cell properties, glioma growth, changes in cancer cell metabolism, and treatment resistance.

HBOT favorably improves oxygen transport to the hypoxic tumor tissues, thereby potentially increasing the sensitivity of tumor cells to antineoplastic treatment. However, while used alone, it does not inhibit tumor growth (33). In contrast, when used as an adjuvant treatment with RT and/or CT, it leads to a significant decrease in tumor size. Initial HBOT can modify the hypoxic microenvironment and impair the stemness-associated properties of cancer cells (51).

Regarding its benefit when used as an adjuvant treatment with TMZ, tumor tissue hypoxia tends to decrease with HBOT, and some hypoxic areas even return to regular oxygen supply. With an increase in oxygen concentration, the tumor tissue becomes more sensitive to CT drugs. In addition, hyperbaric oxygenation also amplifies the cell cycle arresting effect of CT, which might result in an increased affinity of tumor cells for TMZ (33), indicating that for the same drug dose, there is a higher rate of apoptosis. By combining nimustine and HBOT, the expression of inflammatory factors is reduced, and tumor growth is inhibited, indicating that inflammation plays a key role in tumor development (33).

HBOT has been used to treat late-onset RT-related injuries since 1975 (52), being CNS lesions a type 3 recommendation. In 1953, Gray et al. postulated that the primary source of radioresistance was oxygen deficiency (53). As RT's efficacy is based on the tumor tissue's oxygen content, which is why the combination with HBOT amplifies its therapeutic effect (functioning as a radiosensitizer), improving local tumor control and potentially extending survival time.

Using a mouse model of GBM, Wang et al. (54) investigated the effects of HBOT on ROS signaling from transplanted glioma cells, finding that under hyperbaric oxygenation, ROS signaling from glioma and brain cells was diminished. However, their findings raised a new question regarding the pro-oncogenic effects of HBOT on non-cancerous cells, as this therapy appears to produce systemic immunosuppression by inhibiting thymic T-cell maturation (54).

By transplanting gliomas into nude rats, Stuhr et al. (10) concluded that the tumor's hypoxic microenvironment could be altered through hyperoxia. Both normobaric and hyperbaric oxygenation (1.02 ATA vs. 2.04 ATA) inhibit tumor growth after only 4.5 h of treatment (approximately three exposures) (10). Nevertheless, HBOT significantly improves oxygen perfusion, damaging the tumor by inducing apoptosis and reducing its central vascular density.

### Preclinical Hyperbaric Oxygen Therapy Trials

#### Chemoradiotherapy

The anticancer effects of hyperoxia and its action mechanisms are mostly identified in mouse lung cancer (55, 56). To detect the potential mechanism of HBOT in lung tumors, Chen et al. (57) selected, as an *in vivo* model, non-small cell human lung carcinoma A549-cell-transferred severe-combined immunodeficiency mice (SCID). Their results revealed that HBOT improves tissue angiogenesis and tumor hypoxia and increases tumor apoptosis by modifying the tumor's hypoxic microenvironment. The treatment also suppressed tumor growth in murine xenograft tumor models (57). This was the first study to demonstrate that HBOT immediately downregulated endogenous p53 protein, which rebounded to baseline after 20 h of treatment.

Zembrzuska et al. (58) studied the combination of isothiourea derivatives and HBOT, reporting that this regimen appears to be a promising therapeutic approach for malignant glioma, as it reduces the proliferation and viability of *in vitro* GBM cells compared to cells under normoxia and hypoxia.

Brizel et al. (59) also performed *in vivo* studies on rats with implanted mammary adenocarcinoma. These were divided into five groups, posteriorly treated with inhaled normobaric or hyperbaric oxygenation or normobaric or hyperbaric carbogen. Positive results were observed in the groups treated with high-pressure gases (59). Thews and Vaupel (60) conducted a similar study, which tested the influence of normal and hyperbaric oxygenation (pure oxygen vs. carbogen), local hypoxic regions occurrence, and spatial distribution of pO_2_, using the subcutaneously injected DS-sarcoma cell line in rats. Under the pressure of 2.07 ATA, the median pO_2_ was 5-fold higher. Following HBOT, the spatial distribution of pO_2_ profiles showed a nearly absolute elimination of hypoxic regions.

However, isolated HBOT has a limited curative effect and is typically not applied by itself (60).

Stuhr et al. (10) elucidated the effects of hyperbaric oxygenation alone on BT4C rat glioma xenografts *in vivo*. In his study, hyperoxia (both normal and hyperbaric) resulted in a 60% retardation of tumor growth compared to the control group. Although the proliferation rate of cells remained unchanged in the two groups, there were more necrotic areas, 20% more apoptotic cells in control tumors, and no damage to normal tissues (10). More inconsistent results were found with isolated HBOT, probably due to different HBOT procedures and intracranial measurement methods (61, 62).

Regarding the pO_2_ of normal brain tissue, in animal experiments, it has been noted to decrease quickly after HBOT, whereas the pO_2_ in high-grade gliomas falls more slowly after decompression due to the lower rate of oxygen consumption and the reduced blood flow to the tumor (63). Therefore, the pO_2_ within the tumor may remain elevated after decompression for a substantial time, suggesting that HBOT may increase the sensitivity of hypoxic tumor cells, even without increasing injury to the normal brain tissue (64).

Kunugita et al. (65) examined the effect of RT after HBOT on spontaneously arising murine squamous cell carcinomas (SCCVII) (with a radiobiological hypoxic fraction of ~10%) that were subcutaneously transplanted into C3H/He mice using a growth delay assay. They noted a significant SCCVII tumor growth delay in the treated animals within 30 min after HBOT, with the delay time being 1.61 times longer than that after RT alone (66).

#### Chemotherapy

Xie et al. (9), studied the combination of HBOT and nanotemozolomide in glioma using nude mice. Their findings confirm that HBOT is a suitable adjuvant treatment to CT, as the proliferation of tumor tissue was significantly lower when the two therapies were combined (9). Moreover, significant inhibition of tumor growth, weight, and size was also observed, thus supporting the pivotal role HBOT can have in enhancing antitumor agents' efficacy. A decrease in hypoxic fields after HBOT was also verified, adding up to 50% of hypoxic regions modified. Potentiation of the cell cycle arrest effect of the agent was also observed. The authors postulate that the increased tumor oxygen tension improves the therapeutical response to CT.

Accordingly, Lu et al. (67) found that pO_2_ in mice who underwent HBOT combined with nimustine therapy was significantly higher than standard, not only on the healthy brain tissue itself but also on the glioma tumor cells, which were approaching normal brain tissue pressures.

#### Other Treatments

In a mouse model of aggressive metastatic cancer, Poff et al. (68) evaluated the efficacy of metabolic therapies, including the ketogenic diet, ketone supplementation, and hyperbaric oxygenation. *In vitro*, each therapy inhibited the proliferation and viability of tumor cells and decelerated disease progression. Nonetheless, when combined, they potentiated powerful antineoplastic effects by inhibiting metastasis proliferation and doubling the survival time of mice with systemic metastatic disease (69).

### Clinical Hyperbaric Oxygen Therapy Trials

#### In Combination With Chemoradiotherapy

There are scarce data on prospective clinical studies evaluating HBOT in combination with RT or chemoradiation for GBM. Most of the evidence available consists of single-centered trials evaluating RT and HBOT. Nevertheless, findings have shown promising results in patients with high-grade glioma (69, 70).

In 1977, Chang et al. published their findings regarding whether HBOT during RT could improve results in glioma treatment (71). The median OS of the patients in the HBOT group was 38 weeks, compared with 31 weeks for patients in the control group, and survival rates after 18 months were 28 and 10%, respectively.

Kohshi et al. (72), conducted a non-randomized trial that evaluated the feasibility of associating RT with HBOT for 29 high-grade glioma patients. The median OS for patients with and without HBOT was 24 and 12 months, respectively (*p* < 0.05). All patients (*n* = 4) who received RT more than 30 min after decompression had worse outcomes. This constituted the early perception that the timing between modalities was critical to achieving the best outcomes. As no serious side effects were observed in HBOT patients, the authors concluded that irradiation after HBOT appeared to be a valuable treatment for high-grade gliomas. However, irradiation should be administered immediately after decompression (72).

Beppu et al. (69) published their phase II study integrating HBOT and beta-interferon, nimustine hydrochloride (ACNU), and RT (IAR, Interferon-ACNU-Radiotherapy) for the treatment of malignant supratentorial gliomas. Daily RT was completed within 15 min after HBOT. Of the 39 patients, 35 underwent a complete HBOT and IAR therapy schedule. Thirty patients (76.9%) either maintained or increased their Karnofsky Performance Status Scale (KPS) during HBOT/IAR, with a mean duration of 68 days. The combined complete and partial response rates for GBM, anaplastic astrocytoma, and overall general histologies were 50, 30, and 43%, respectively. Median time to progression (TTP) for GBM, anaplastic astrocytoma, and overall patients was 38, 56, and 43 weeks, respectively. The authors suggested that HBOT/IAR therapy could be applied to selected patients with poor prognostic factors due to its short treatment period, acceptable toxicity profile, and identical response when compared to patients with good prognostic factors (69).

Ogawa et al. reported the long-term results of a phase II trial for daily conventionally fractionated RT (60 Gy/30 fractions) 15 min after HBOT with multiagent CT (procarbazine, nimustine, and vincristine) in patients with high-grade gliomas. All patients were able to complete the RT immediately after HBOT. The median OS of 39 patients with GBM was 17.2 months. However, acute toxicity developed in almost 48 % of patients (70, 72).

Kohshi et al. (73) also investigated the combination of HBOT with fractionated stereotactic RT (FSRT) in a small cohort of previously irradiated patients with recurrent gliomas. The median OS of patients treated with this protocol was 19 and 11 months for anaplastic astrocytoma and GBM patients, respectively (73).

In another Japanese study, Yahara et al. (74) studied IMRT boosts after HBOT concurrent with TMZ-based CT. Of note, the authors estimated a biologically effective dose (BED) of 85.8 Gy10 for the high-dose clinical target volume (CTV). Previous studies have reported that dose escalation of RT for GBM above 60 Gy using conventional RT had limited success. The total BED of 85.8 Gy10 was relatively low compared to other trials. The first site of progression was locally in 58% of the patients with GBM, and grade ≥3 toxicity, such as brain radionecrosis, was not observed. The median OS was 22.1 months, which was promising considering a cohort of patients with GBM (75).

Arpa et al. (6) published their experience with HBOT and RT in a pilot study for recurrent high-grade glioma patients. In their small series of nine patients, RT was administered in daily 5-Gy fractions for 3–5 consecutive days. Each fraction was delivered within 1 h after HBOT. The disease control rate 3 months after HBOT-RT was 55.5% (5 patients), the median PFS for all patients was 5.2 months (95% CI: 1.34-NE), while 3- and 6-month PFS was 55.5% (95% CI: 20.4–80.4) and 27.7% (95% CI: 4.4–59.1), respectively. The median OS was 10.7 months (95% CI: 7.7-NE). No acute or late neurologic toxicity grade >2 was observed in 88.88% of patients, although one developed grade 3 radionecrosis. Despite the small cohort of selected patients, the paper poses a convenient short course alternative to systemic therapies for patients who cannot or refuse to undergo such treatments in this late stage of disease. To our knowledge, this is the only recruiting trial in this setting on ClinicalTrials.gov (6).

### Studies' Limitations and New Perspectives

Altogether, the clinical studies enrolled over 200 patients with gliomas who received HBOT shortly before each RT fraction (Table 1).

**Table 1:** Summary of preclinical or clinical trials present in the literature, referring to the use of hyperbaric oxygen therapy in the treatment of gliomas and tumor cells, as well as in the carcinogenesis process.

	**References**	**Tumor type**	**Population**	**Protocol**	**Results**
Preclinical	Chen et al. (57)	Non-small cell human lung carcinoma	C.B-17 SCID	After being injected with human lung carcinoma A549 cell line, the subjects were divided into two groups. The experimental group was exposed to 98% oxygen at 2.5 ATA for 90 min daily, 5 days per week, during 2 weeks.	- Tumor growth was suppressed 14 (*p* < 0.01) and 28 (*p* < 0.01) days after HBOT exposure; - Tumor hypoxia was improved 14 (*p* < 0.05) and 28 (*p* < 0.05) days after HBOT exposure; - HBOT induced tumor apoptosis; - HBOT increased the expression of CD31 14 and 28 days after exposure; - HBOT downregulated p53 protein and increased HIF-1α only in A549 cells.
	Zembrzuska et al. (58)	Human GBM	Cultures of human GBM T98G cell line	Cells were cultured in gaseous mixtures with various oxygen contents. A modified isothiourea derivative (ZKK-3) was added in different concentrations to the experimental groups.	- The combination of HBOT and 25 and 50 μM ZKK-3 decreased GBM cell proliferation rate; - The combination of HBOT and ZKK-3 significantly reduced GBM cell viability; - HBOT alone significantly decreases HIF-1α expression.
	Thews and Vaupel (60)	DS-sarcoma cell line	Sprague–Dawley rats	After tumor implantation, subjects were submitted to different oxygen concentrations (either inhaling 100 or 95% oxygen) and environmental pressures (either 1 or 2 ATA).	- In a hyperbaric environment (e.g., 2 ATA), tumor oxygenation homogeneously increased throughout the whole mass; - HBOT eliminated tumor hypoxia.
	Stuhr et al. (10)	Glioma	Athymic nude rats	After subcutaneous injection of BT4C cell line, subjects were divided into groups, which were exposed to different oxygen concentrations and pressures. The experimental group submitted to HBOT was treated 3 times per day, for 90 min, during 3 non-consecutive days.	- In the hyperoxic groups, tumor growth decreased; - Hyperoxic treatment induced tumor apoptosis (*p* < 0.001) and necrosis; - Hyperoxia did not enhance cell proliferation; - Tumor vessel diameter diminished in hyperoxia (*p* < 0.01); - Hyperoxia induced pro-apoptotic markers and suppressed anti-apoptotic and pro-angiogenic genes; - HIF-2α was repressed by hyperoxia.
	Kunugita et al. (65)	Gliosarcoma and squamous cell carcinomas	Fisher 344 rats and C3H/He mice	After tumor inoculation, the subjects were exposed to 100% oxygen, at 2.0 ATA, for 60 min. Irradiation of the tumor-containing limb was immediately performed 5 min after decompression in each experimental group.	- Combination of HBOT and RT significantly decreased tumor growth in the squamous cell carcinoma group (*p* < 0.01); - Tumor reduction was significantly enhanced in the groups which combined HBOT and RT.
	Sümen et al. (64)	N/A	Sprague-Dawley rats	The anti-inflammatory activity of HBOT was tested in comparison to diclofenac in response to carrageenan-induced acute inflammation. The experimental group was submitted to HBOT for 90 min at 2.4 ATA.	- Both diclofenac and HBOT greatly reduced oedema in rat paws; - Combination of HBOT and diclofenac had a greater effect than monotherapy in reducing oedema.
	Brizel et al. (59)	Mammary adenocarcinoma	Fischer 344 rats	After subcutaneous tumor implantation, the subjects were exposed to different concentrations of oxygen under various environmental pressures. HBOT was performed at 3 ATA.	- Both low pO_2_ tumor values and median pO_2_ tumor values significantly improved after 5 min of HBOT. This effect was sustained after 20 and 25 min, respectively; - HBC did not improve tumor oxygenation more than HBOT; - Sympathetic blockade with bretylium prior to HBC exposure significantly improved oxygenation more than HBC alone.
	Jamieson and van den Brenk (63)	N/A	Canberra black or Wistar hooded rats and guinea pigs	Electrodes were inserted into the brain or subarachnoid space of the subjects 24 h prior to each experimental run. The hyperbaric chamber was pressurized until a maximum of 6 ATA.	- Cerebral oxygen tension increased abruptly in pressures above 1–2 ATA; - Compared to pure oxygen, tissue pO_2_ had higher values when carbogen was inhaled for pressures >2 ATA.
	Xie et al. (9)	Rat glioma	BALB/c/nude mice (*in vivo*) and rat glioma C6 cell line cultures (*in vitro*)	Subjects and cells were exposed to different concentrations of oxygen and pressures after treatment with TMZ or nanotemozolomide.	- Combination of HBOT with TMZ or nanotemozolomide significantly inhibited tumor growth; - Combination of HBOT with nanotemozolomide significantly suppressed cell proliferation; - After HBOT, more than 50% of hypoxic tumor regions were modified; - The decrease in cell viability was higher after HBOT; - HBOT potentiated the cell cycle arrest effect of TMZ.
	Lu et al. (67)	Human glioma	Nude mice expressing EGFP	After injecting the subjects with human glioma stem/progenitor cell line SU3, these were divided into groups exposed to HBOT and/or nimustine. HBOT occurred at 2.6 ATA for 90 min, daily, over 21 days.	- Combination of HBOT and nimustine deaccelerated tumor growth rate more rapidly; - Combination of HBOT and nimustine significantly reduced tumor volume (*p* < 0.05); - In combination, HBOT and nimustine had a synergistic effect; - Combination of HBOT and nimustine reduced the degree of necrosis and inflammatory cell infiltration; - Inflammatory factors were significantly reduced in HBOT and HBOT and nimustine groups.
	Poff et al. (68)	Brain tumor	VM/Dk inbred mice	After subcutaneous tumor implantation, subjects were divided into groups with different diets and hyperbaric exposure. Those submitted to HBOT, completed sessions of 100% oxygen at 2.5 ATA, 3 times per week.	- Combination of HBOT and metabolic therapy decreased tumor burden, as well as metastatic spread; - Both metabolic therapy and its combination with HBOT prolonged survival; - Subjects exposed to low glucose diets and HBOT had lower tumor growth; - Combination of HBOT with low glucose and ketone supplementation decreased tumor cell viability.
Clinical	Arpa et al. (6)	Recurrent high-grade glioma	9 patients	Patients were irradiated daily, up to 60 min after HBOT.	- After 3 months, disease control rate was 55.5%; - Overall median PFS was 5.2 months; - Median OS was 10.7 months; - 88.88% of patients developed acute or late neurologic toxicity grade >2.
	Yahara et al. (74)	Primary glioblastoma	24 patients with newly diagnosed GBM, treated with postoperative RT or post-biopsy RT	Patients underwent IMRT 15 min after a HBOT session of 60–90 min at 2.0 ATA. All patients were treated with CT during the course of RT.	−58% of patients firstly developed local progression; - Median OS was 22.1 months; - Grade ≥3 toxicity was not observed.
	Ogawa et al. (70)	High-grade glioma	40 patients	Patients were submitted to daily conventionally fractionated RT, 15 min after HBOT, with multiagent CT.	−57% of patients had an objective response (either complete or partial remission); - Median TTP in GBM was 12.3 months; - GBM had a median OS of 17.2 months; - 48% of patients developed treatment toxicity.
	Kohshi et al. (73)	Recurrent glioma	25 patients, previously irradiated	Patients who had previously received RT and CT were submitted to Gamma FSRT after HBOT; HBOT was performed at 2.5 ATA for 60 min.	- Anaplastic astrocytoma had a median OS of 19 months; - GBM had a median OS of 11 months; - Overall, 28% of patients underwent additional surgical procedures 8.4 months after FSRT.
	Beppu et al. (69)	Malignant supratentorial glioma	35 patients	Daily RT was completed within 15 min after HBOT, in combination with interferon-beta and nimustine administration.	−76.9% either maintained or increased KPS during treatment; - GBM had a combined complete and partial response rate of 50%; - Anaplastic astrocytoma had a combined complete and partial response rate of 30%; - Overall, patients had a median TTP of 43 weeks; - GBM had a median TTP of 38 weeks; - Anaplastic astrocytoma had a median TTP of 56 weeks.
	Kohshi et al. (72)	Recurrent glioma	29 patients, previously irradiated	Patients underwent RT 15 to 30 min after HBOT, daily.	- In the HBOT group, median OS was 24 months; - Patients with anaplastic astrocytoma treated with this protocol had a median OS of 19 months; - Patients with GBM had treated with this protocol had a median OS of 11 months; - There were no seizures, radiation necrosis or other adverse effects reported in the HBOT group.
	Chang et al. (71)	Glioma	80 patients, previously untreated	Patients were irradiated under HBOT.	- Median OS of the patients in the HBOT group was 38 weeks - After 18 months, the survival rate in the HBOT group was 28%.

*ATA, atmosphere absolute; CT, chemotherapy; EGFP, enhanced green fluorescent protein; FSRT, fractionated stereotactic radiotherapy; GBM, glioblastoma; HBC, hyperbaric carbogen; HBOT, hyperbaric oxygen therapy; IMRT, intensity-modulated radiotherapy; KPS, Karnofsky Performance Status Scale; N/A, non-applicable or non-available; OS, overall survival; pO_2_, partial pressure of oxygen; RT, radiotherapy; SCID, severe-combined immunodeficiency mice; TMZ, temozolomide; TTP, time to progression*.

The challenge of high-level evidence is multifactorial, considering the availability of HBOT and RT at the same institution, logistics to timely delivered RT, coordination between centers, and frailty of the targeted population to trial accrual candidates for a more robust trial. Another major obstacle to high-quality evidence is the simulation of a therapeutic session in a hyperbaric chamber. According to Lansdorp and van Hulst (75), the best approach to replicate the experience is to employ lower pressure and a gaseous mixture with 21% oxygen. Currently, this is considered the paramount approach to designing the sham-controlled arm (75).

Nevertheless, the findings from both the single- and double-arm studies indicated improvement in some outcomes (e.g., OS, PFS, TTP, and response rate) with HBOT and RT. Although reported toxicity included leukopenia, anemia, thrombocytopenia, fever, anorexia, and gastrointestinal symptoms like nausea, vomiting, or liver dysfunction, these reports cannot be attributed solely to HBOT. Patients with severe MEB were also uncommon (6, 70, 74, 76–78). So far, data favor further research on the clinical use of HBOT and RT for high-grade gliomas.

Thus, even though the available literature supports greater integration of HBOT into the treatment options, this should be preceded by precise planning and delivery in predetermined appropriate clinical settings.

Technological advances in Medical Physics, Machine Engineering, and Bioinformatics also bring new opportunities for enhancing RT's therapeutic index. A promising solution to fulfill the strict time interval between HBOT and RT could be the use of a device capable of performing both therapies, such as the one developed by HAUX-LIFE-SUPPORT GmbH for research at the University of Mainz (79).

Regarding radiation treatment, proton beam therapy (PBT) is a particle radiation therapy that uses protons instead of photons. Proton beams have the physical property (described as the Bragg's Peak) to deliver charged particles with a finite, energy-dependent range in tissue that can be adjusted to match the depth of the target. These results in a steep dose fall-off at the end of the particle path, allowing for better sparing of normal tissue.

Clinically, this offers an opportunity to improve RT for primary gliomas by reducing or eliminating the exposure of healthy tissues. This is of increasing interest, particularly in children.

Preventing irradiation of radiosensitive structures (e.g., hippocampus and cerebral cortex) and reducing the overall irradiated brain volume may improve quality of life endpoints, including neurocognitive dysfunctions and endocrine abnormalities. However, in spite of its dosimetric advantage and reported safety profile, PBT is not as widely available as photon-based RT, requiring greater investment.

A potential margin for dose escalation with PBT in GBM is an area of future research (80–85). In a National Cancer Data Base (NCDB) analysis, PBT was associated with improved outcomes in OS in adult patients either with low-grade or high-grade gliomas. Yet, its retrospective nature and inability to account for all potential confounding factors limit definitive conclusions. Other particle beams such as neutron and carbon ions are also of interest in glioma treatment investigation (85).

Currently, ongoing investigations are evaluating the use of PBT for gliomas. A Mayo Clinic trial studies the efficacy of 6-Fluoro-(18F)-l-3,4-dihydroxyphenylalanine-PET/MRI scan (18F-DOPA-PET/MRI) in the imaging of elderly patients with newly diagnosed GBM when planning for a short course of PBT. Using 18F-DOPA-PET scans and MRI scans may provide the radiation oncologist information on tumor vs. normal tissue, potentially contributing to a more accurate plan for radiation delivery.

FLASH RT uses ultrahigh-dose rates several times higher than any conventional dose rates used in RT or radiosurgery, delivering a RT session in nanoseconds. As a result, it generates a phenomenon known as the “FLASH effect.”

FLASH RT can overcome the limitations of nearby sensitivity of normal tissue and allow an increased radiation dose delivered to targets while keeping the toxicity of surrounding healthy tissues low (84–86). Oxygen plays a key role in the underlying biological mechanism resulting in the FLASH effect. Studies described ultrahigh-dose rate radiation could deplete local oxygen and induce a short-term protective hypoxic environment to the surrounding normal healthy tissues, increasing radioresistance. Although the oxygen depletion hypothesis is the most popular current explanation for the FLASH effect, other factors may play a role, including changes in ROS and redox chemistry between normal and tumor cells following FLASH dose rates, as well as immune responses and tumor microenvironment. The role played by this factor also requires further research. Remarkably, a first-in-human report has been published about a patient with CD30+T-cell cutaneous lymphoma treated using FLASH RT with electron beam therapy (84). FLASH RT still must overcome several technical limitations, but its property of inducing radioresistance to normal tissue can potentially change the future of clinical cancer treatment (86–89).

Lately, several radiosensitizers have been evaluated to reduce chronic and acute tumor hypoxia. For the past two decades, the DAHANCA group has been studying the integration of nimorazole in the standard therapeutic plan for head and neck cancers with positive results (90–94). To date, there is little knowledge of its use in high-grade glioma. One study evaluated the development of a bioresorbable layer for controlled TMZ and nimorazole release for palliative GBM treatment (95). However, the use of nimorazole (as an anti-hypoxia agent during RT) for high-grade gliomas remains to be clarified.

## Final Considerations

Survival of GBM patients depends on local disease control, as most will recur in proximity to the primary tumor site (6). Despite the endeavors and achievements made in treating GBM during the past decades, the prognosis remains poor, and long-term survival is rare.

Since the 1950s, researchers have assumed that oxygen is required to induce DNA damage and hypoxic adaptation is particularly restrictive to successful treatment of many solid tumors, including GBM. There are currently emerging data reporting that GBM patients undergoing combined RT and/or CT with HBOT are promising in terms of efficacy and safety. Presently, accurate quantification of the clinical efficacy of this therapeutical approach is still lacking. Further prospective and randomized studies are necessary to validate HBOT's effectiveness in standard clinical practice.

## Data Availability Statement

The original contributions presented in the study are included in the article/supplementary material, further inquiries can be directed to the corresponding author/s.

## Author Contributions

DAC conceived and designed this review. DAC and MS-A performed the acquisition, analysis, and interpretation of the data. Regarding the writing and detailed literature review, these were distributed as follows: EO—glioblastoma. EN and GF—hypoxia and radioresistance. DAC and MS-A—hypoxia and chemoresistance. AT, PD, GB, and CA—HBOT. DAC and MS-A—HBOT as a hypoxia reverser treatment. EN, GF, MS-A, and DAC—preclinical and clinical HBOT trials and the studies' limitations and future perspectives. Manuscript supervision was conducted by DAC and MS-A. All authors contributed to the article and approved the submitted version.

## Conflict of Interest

The authors declare that the research was conducted in the absence of any commercial or financial relationships that could be construed as a potential conflict of interest.

## Publisher's Note

All claims expressed in this article are solely those of the authors and do not necessarily represent those of their affiliated organizations, or those of the publisher, the editors and the reviewers. Any product that may be evaluated in this article, or claim that may be made by its manufacturer, is not guaranteed or endorsed by the publisher.
